
JOURNAL INFORMATION
==============================
NLM Title Abbreviation: J Vet Intern Med
Journal ID: J Vet Intern Med
NLM Journal ID: 8708660
Journal ID: JVIM

Journal of Veterinary Internal Medicine

ISSN: 0891-6640
EISSN: 1939-1676
Publisher: Oxford University Press

ARTICLE INFORMATION
==============================
PMCID: PMC11474594
DOI: 10.1111/jvim.16756
Article ID: JVIM16756
Article version: 1
Subjects: Issue Information, Issue Information

Issue Information

Electronic publication date: 2024 Mar 13
Publication date: 2024 Mar-Apr
Volume: 38
Issue: 2
First page: 865
Last page: 871
Copyright: © 2024 The Authors. Journal of Veterinary Internal Medicine published by Wiley Periodicals LLC on behalf of American College of Veterinary Internal Medicine.
License: This is an open access article under the terms of the http://creativecommons.org/licenses/by/4.0/ License, which permits use, distribution and reproduction in any medium, provided the original work is properly cited.
License URL: https://creativecommons.org/licenses/by/4.0/

Figure count: 0
Table count: 0
Page count: 1

source-schema-version-number: 2.0
cover-date: March/April 2024
details-of-publishers-convertor: Converter:WILEY_ML3GV2_TO_JATSPMC version:6.4.9 mode:remove_FC converted:15.10.2024

==============================

Volume 38, Number 2 March/April 2024

### JOURNAL OF VETERINARY INTERNAL MEDICINE

Open Access

ACVIM
American College of Veterinary Internal Medicine

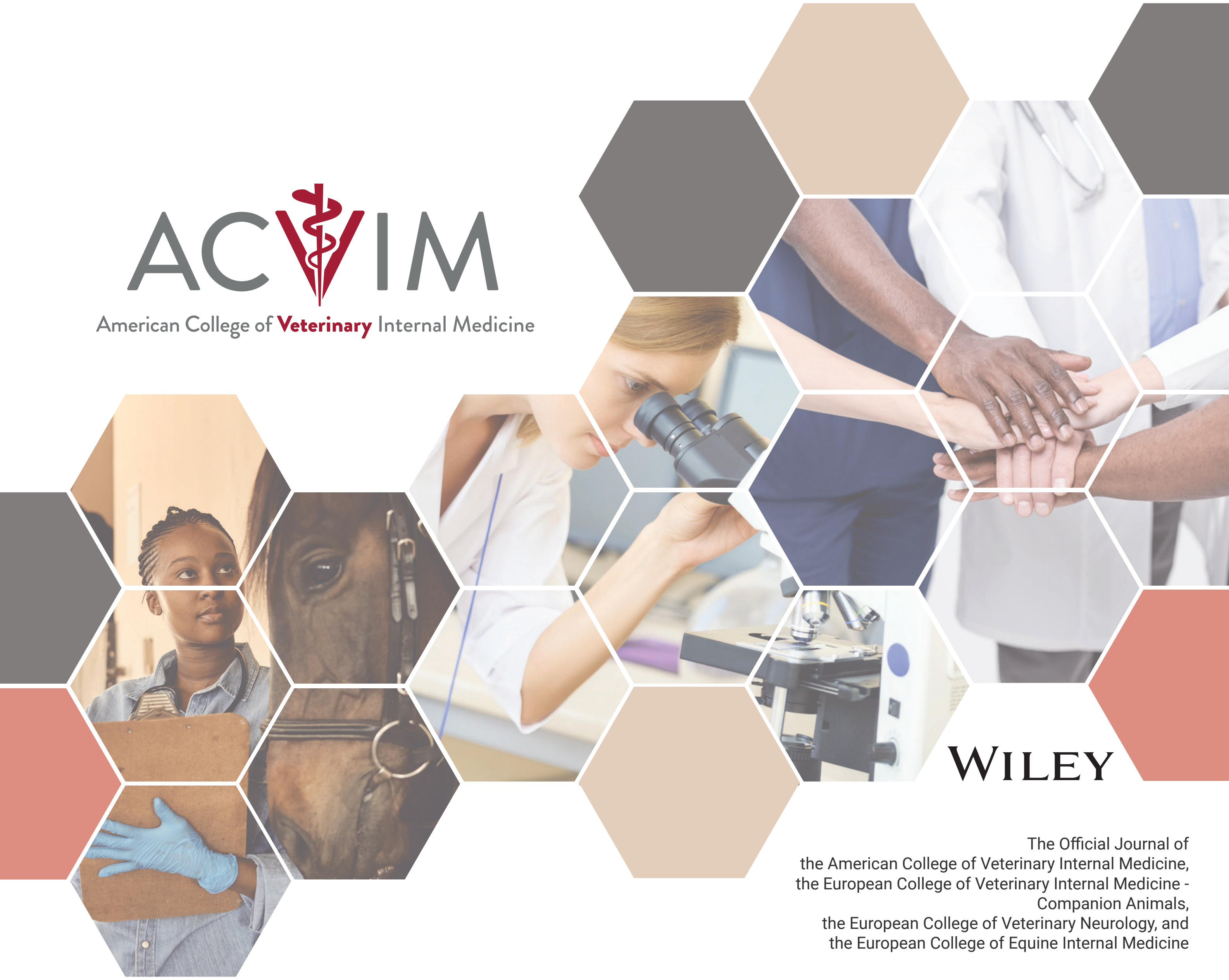

WILEY

The Official Journal of
the American College of Veterinary Internal Medicine,
the European College of Veterinary Internal Medicine -
Companion Animals,
the European College of Veterinary Neurology, and
the European College of Equine Internal Medicine

### Journal of Veterinary Internal Medicine

Open Access

ACVIM American College of Veterinary Internal Medicine

The Official Journal of the American College of Veterinary Internal Medicine,
the European College of Veterinary Internal Medicine - Companion Animals, the European College of Veterinary Neurology, and the European College of Equine Internal Medicine

**MISSION STATEMENT**

The mission of the *Journal of Veterinary Internal Medicine* is to advance veterinary medical knowledge and improve the lives of animals by publication of authoritative scientific articles of animal diseases.

**Volume 38, Number 2** **March/April 2024**

EDITORIAL

872 **Scientific writing and editorial policies and procedures of the Journal of Veterinary Internal Medicine**
*Stephen P. DiBartola and Kenneth W. Hinchcliff*

CONSENSUS STATEMENT

878 **ACVIM consensus statement on diagnosis and management of urinary incontinence in dogs**
*Allison Kendall, Julie K. Byron, Jodi L. Westropp, Joan R. Coates, Shelly Vaden, Chris Adin, Garrett Oetelaar, Joe W. Bartges, Jonathan D. Foster, Larry G. Adams, Natasha Olby, and Allyson Berent*

SMALL ANIMAL

Standard Articles

*Cardiology*

904 **Relationship between syringomyelia and myxomatous mitral valve disease in Cavalier King Charles spaniels**
*Maiken B. T. Bach, Camilla L. Stougaard, Maria S. Thøfner, Maria J. Reimann, Ulrik Westrup, Jørgen Koch, Merete Fredholm, Torben Martinussen, Mette Berendt, and Lisbeth H. Olsen*

913 **Effect of amlodipine on the circulating renin-angiotensin-aldosterone system in healthy cats**
*Tatiana M. Garcia Marrero, Jessica L. Ward, Melissa A. Tropf, Agnes Bourgois-Mochel, Emilie Guillot, Oliver Domenig, Lingnan Yuan, Debosmita Kundu, and Jonathan P. Mochel*

922 **Automated echocardiographic left ventricular dimension assessment in dogs using artificial intelligence: Development and validation**
*Catherine C. Stowell, Valeria Kallassy, Beth Lane, Jonathan Abbott, Kieran Borgeat, David Connolly, Oriol Domenech, Joanna Dukes-McEwan, Luca Ferasin, Josefa Fernández Del Palacio, Chris Linney, Jose Novo Matos, Ilaria Spalla, Nuala Summerfield, Tommaso Vezzosi, James P. Howard, Matthew J. Shun-Shin, Darrel P. Francis, and Virginia Luis Fuentes*

*Endocrinology*

931 **Assessment of the likelihood of hypothyroidism in dogs diagnosed with and treated for hypothyroidism at primary care practices: 102 cases (2016-2021)**
*Victoria Travail, Carolina Fernandez Sanchez, Jose M. Costo, Nicola Valentine, Megan Conroy, Venessa Lee, Dimitrios Bouziopoulos, Kathryn Bateman, Emma Gatehouse, Judith Cruzado-Perez, Danica Pollard, Valerie Lamb, Florence Juvet, and Darren Kelly*

942 **Hypothalamic-pituitary-adrenal axis recovery after intermediate-acting glucocorticoid treatment in client-owned dogs**
*Francesca Del Baldo, Andrea Corsini, Antonio Maria Tardo, Alessandro Tirolo, Ada Sapignoli, Michele Tumbarello, Kateryna Vasylyeva, and Federico Fracassi*

951 **A retrospective study of hydrocortisone continuous rate infusion compared with administration of dexamethasone boluses in dogs with adrenal crisis**
*Lara Brunori, Olivia X. Walesby, Daniel H. Lewis, and Alisdair M. Boag*

960 **Serum symmetric dimethylarginine in older dogs: Reference interval and comparison of a gold standard method with the ELISA**
*Sofie Marynissen, Greet Junius, Evi Van den Steen, Lisbeth Patteet, Luc Duchateau, Siska Croubels, Sylvie Daminet, and Dominique Paepe*

971 **Clustering analysis of lipoprotein profiles to identify subtypes of hypertriglyceridemia in Miniature Schnauzers**
*Nicole M. Tate, Punyamanee Yamkate, Panagiotis G. Xenoulis, Jörg M. Steiner, Erica L. Behling-Kelly, Aaron K. Rendahl, Yu-An Wu, and Eva Furrow*

980 **Development of a requirement for exogenous insulin treatment in dogs with hyperglycemia**
*Angielee DiNinni and Rebecka S. Hess*

987 **Serial monitoring of pancreatic lipase immunoreactivity, C-reactive protein, abdominal ultrasonography, and clinical severity in dogs with suspected pancreatitis**
*Leslie Mitchell, Sichao Wang, Jody Lawver, and Harry Cridge*

995 **Candidate circulating microRNA biomarkers in dogs with chronic pancreatitis**
*Susan K. Armstrong, Robert W. Hunter, Wilna Oosthyuzen, Maciej Parys, Adam G. Gow, Silke Salavati Schmitz, James W. Dear, and Richard J. Mellanby*

*Gastroenterology*

1005 **Clinicopathological findings, treatment, and outcome in 60 cats with gastrointestinal eosinophilic sclerosing fibroplasia**
*Petra Černá, Cristobal Lopez-Jimenez, Kenjiro Fukushima, Ko Nakashima, Taisuke Nakagawa, Fiona Adam, Anna Groth, Andrew Denning, Nicolas Israeliantz, and Danièlle A. Gunn-Moore*

*Hematology*

1013 **The effect of ε-aminocaproic acid on blood product requirement, outcome and thromboelastography parameters in severely thrombocytopenic dogs**
*Johanna Wolf, Laura K. Ruterbories, Ian Handel, and Bernie Hansen*

1022 **A prospective cohort study to identify clinical diagnostic and prognostic markers of primary immune thrombocytopenia in dogs**
*Marjory B. Brooks, Robert Goggs, Amelia H. Frye, Jessica Armato, Marnin Forman, Julia Hertl, Michael Koch, John P. Loftus, John Lucy, Brandi Mattison, Julia Merriam, Sarah Shropshire, Laura Van Vertloo, Austin Viall, and Dana N. LeVine*

1035 **Comparison of timing of relapse in dogs with nonassociative immune-mediated hemolytic anemia, thrombocytopenia, or polyarthritis**
*Richard Sparrow, James W. Swann, and Barbara Glanemann*

*Hepatology*

1043 **Diagnostic and predictive ability of hyperbilirubinemia severity in cats: A multicenter retrospective study**
*Xavier Salord Torres, Kamalan Jeevaratnam, Imogen Schofield, Samantha Taylor, Jennifer Stallwood, Menai Heyes, Daniel Hughes, and Pieter Defauw*

*Nephrology/Urology*

1051 **Retrospective evaluation of 22 dogs with leptospirosis treated with extracorporeal renal replacement therapies (2018-2021)**
*Antonia Da Fonseca Ioannou, Carolyn Tai, Mary Anna Labato, and Emmanuelle M. Butty*

1060 **Evaluation of a urine dipstick protein to urine specific gravity ratio for the detection of proteinuria in dogs and cats**
*Michael Barchilon, Nichole Perez-Nieves, and Jean-Sébastien Palerme*

1068 **Correlation between urine anion gap and urine ammonia-creatinine ratio in healthy cats and cats with kidney disease**
*Alyssa R. Berman, Andrew J. Specht, Rebeca A. Castro, Kirsten L. Cooke, Shir Gilor, and Autumn N. Harris*

*Neurology*

**1074** **Serum concentrations of complement C3 and C4 in dogs with idiopathic epilepsy**
*Seonggweon Kang, Yoonhoi Koo, Taesik Yun, Yeon Chae, Dohee Lee, Hakhyun Kim, Mhan-Pyo Yang, and Byeong-Teck Kang*

**1083** **Comparison of D-dimer concentration and thromboelastography for diagnosis of cerebrovascular accidents in dogs: A retrospective study**
*Elizabeth DiPaola, Starr Cameron, Helena Rylander, Natalia Zidan, and Scott Hetzel*

**1092** **A retrospective study of the efficacy of zonisamide in controlling seizures in 57 cats**
*Dylan M. Djani, Michael Liou, Srikanth Aravamuthan, Vivian Lau, and Starr Cameron*

**1101** **Intraobserver and interobserver agreement of 8 segmental reflexes in healthy dogs**
*Bryan Chiang, Gabriel Garcia, Francesco Leverone, Jorge A. Hernandez, and Sheila Carrera-Justiz*

**1111** **Clinical and imaging findings in dogs with nerve root signature associated with cervical intervertebral disc herniation**
*Jordan Schachar, Alan Bocage, Nathan C. Nelson, Peter J. Early, Christopher L. Mariani, Natasha J. Olby, and Karen R. Muñana*

**1120** **Sensorineural deafness in purebred white Devon Rex cats**
*Annemarie Kortas, Andrzej Pomianowski, Malgorzata Kolecka, and Liliana Rytel*

*Oncology*

**1127** **Reason for euthanasia in dogs with urothelial carcinoma treated with chemotherapy or radiation therapy or both: A retrospective observational study**
*Charly McKenna, Valerie J. Poirier, Michelle L. Oblak, Stephanie Nykamp, and Anthony J. Mutsaers*

*Respiratory*

**1135** **Evaluation of risk factors for sleep-disordered breathing in dogs**
*Iida Niinikoski, Sari-Leena Himanen, Mirja Tenhunen, Mimma Aromaa, Liisa Lilja-Maula, and Minna M. Rajamäki*

Case Reports

*Cardiology*

**1146** **Medial strabismus (esotropia) at rest associated with contralateral paramedian thalamic ischemic infarction in 2 dogs**
*Theofanis Liatis, Ioannis N. Plessas, Holger Volk, and Danielle Whittaker*

*Endocrinology*

**1152** **Spontaneous remission and relapse of diabetes mellitus in a male dog**
*Mariola B. Rak, Chen Gilor, Stijn J. M. Niessen, and Eva Furrow*

**1157** **Acute hypernatremia and hypocalcemia after oral sodium phosphate administration to a dog**
*Julia VanDerslice and Maureen Carroll*

*Genetics*

**1160** **Association of a novel *dystrophin (DMD)* genetic nonsense variant in a cat with X-linked muscular dystrophy with a mild clinical course**
*Harunobu Muto, Yoshihiko Yu, James K. Chambers, Lyndon M. Coghill, Yasuharu Nakamura, Kazuyuki Uchida, and Leslie A. Lyons*

**EQUINE**

Standard Articles

*Cardiology*

1167 **Prevalence and severity of exercise-induced pulmonary hemorrhage in 2-year-old Thoroughbred racehorses and its relationship to performance**
*Sierra Shoemaker, Yuan Wang, Debra Sellon, Jenifer Gold, Allison Fisher, Julia Bagshaw, Renaud Leguillette, Macarena Sanz, and Warwick Bayly*

*Endocrinology*

1177 **Effect of phenylbutazone on insulin secretion in horses with insulin dysregulation**
*Kate L. Kemp, Jazmine E. Skinner, and François-René Bertin*

*Hematology*

1185 **Effect of leukoreduction on the metabolism of equine packed red blood cells during refrigerated storage**
*Arianna Miglio, Francesca Rocconi, Valentina Cremonini, Angelo D'Alessandro, Julie A. Reisz, Mark Maslanka, Ian S. Lacroix, Giorgio Tiscar, Morena Di Tommaso, and Maria T. Antognoni*

1196 **Association of the neutrophil-lymphocyte ratio with outcome in sick hospitalized neonatal foals**
*Amanda N. Samuels, Ahmed M. Kamr, Stephen M. Reed, Nathan M. Slovis, Laura D. Hostnik, Teresa A. Burns, and Ramiro E. Toribio*

*Neurology*

1207 **Measurement of 8-hydroxy-2′-deoxyguanosine in serum and cerebrospinal fluid of horses with neuroaxonal degeneration and other causes of proprioceptive ataxia**
*Megan Palmisano, Jeaneen Kulp, Susan Bender, Darko Stefanovski, Mary Robinson, and Amy Johnson*

*Respiratory*

1214 **A CONSORT-guided, randomized controlled clinical trial of nebulized administration of dexamethasone and saline on lower airway cytokine mRNA expression in horses with moderate asthma**
*Stephanie Bond and Renaud Léguillette*

1224 **The efficacy of N-acetylcysteine in decreasing airway inflammation and mucus accumulation in horses with 18 hours of head confinement**
*Hamid Tavanaeimanesh, Zahra Alinia, Sirous Sadeghian Chaleshtori, Hamidreza Moosavian, Zeinab Mohebi, and Mojtaba Daneshi*

**FOOD ANIMAL**

Standard Article

*Neurology*

1232 **Comparison of atlantoaxial and lumbosacral cerebrospinal fluid centesis techniques in South American camelids**
*Ester Malmström, Robert C. Cole, Erik H. Hofmeister, Jere K. Stern, and Thomas Passler*

**ABSTRACT**

1240 **16th European College of Equine Internal Medicine (ECEIM) Congress**

**LIST OF REVIEWERS**

1263 **Journal of Veterinary Internal Medicine manuscript reviewers who critiqued in the 2023 calendar year**

### Journal of Veterinary Internal Medicine

Open Access

ACVIM American College of Veterinary Internal Medicine

The Official Journal of the American College of Veterinary Internal Medicine,
the European College of Veterinary Internal Medicine - Companion Animals, the European College of Veterinary Neurology,
and the European College of Equine Internal Medicine

### EDITORIAL BOARD

Kenneth W. Hinchcliff, BVSc, PhD, DACVIM
Editor-in-Chief
The University of Melbourne
Stephen P. DiBartola, DVM, DACVIM
Editor-in-Chief
The Ohio State University
Joshua Stern, DVM, PhD, DACVIM
Associate Editor - Cardiology
North Carolina State University
Jose Novo Matos, DVM, PhD, DECVIM-CA, AFHEA, MRCVS
Associate Editor - Small Animal Internal Medicine, Cardiology
University of Cambridge
Mark Stevenson, MVSc, PhD, MANZCVSc
Consulting Editor for Experimental Design & Statistics
The University of Mebourne
George Moore, DVM, MS, PhD, DACVIM
Consulting Editor for Experimental Design & Statistics
Purdue University
Kristopher J. Hughes, BVSc (Hons 1), FANZCVS, DECEIM, Associate ACVIM
ECEIM Associate Editor - Large Animal Internal Medicine (Equine)
Charles Sturt University
Linda Kidd, DVM, PhD, DACVIM
Associate Editor - Small Animal Internal Medicine (Infectious Disease)
Linda Kidd Veterinary Internal Medicine Consulting
M. Julia B. Felippe, MV, MSc, PhD, DACVIM
Associate Editor - Large Animal Internal Medicine (Equine)
Cornell University
Shelly L. Vaden, DVM, PhD, DACVIM
Associate Editor - Small Animal Internal Medicine (Nephrology/Urology)
North Carolina State University
Michael Willard, DVM, DACVIM
Associate Editor - Small Animal Internal Medicine
Texas A&M University
Andrea Tipold, DVM, DECVN
ECVN Associate Editor - Neurology
School of Veterinary Medicine Hannover
Robert Rebhun, DVM, PhD, DACVIM
Associate Editor - Hematology/Oncology
University of California, Davis
Eric Zini, PD, PhD, DECVIM-CA
ECVIM-CA Associate Editor - Small Animal Internal Medicine (Endocrinology)
University of Zurich & Istituto Veterinario di Novara & University of Padua
Munashe Chigerwe, BVSc, MPH, PhD, DACVIM
Associate Editor - Large Animal Internal Medicine (Food & Fiber)
University of California, Davis
Lauren Trepanier, DVM, PhD, DACVCP, DACVIM
Associate Editor – Small Animal Internal Medicine (Pharmacology, Pharmacokinetics, Toxicology, and Therapeutic Trials)
University of Wisconsin
Valerie Parker, DVM, DACVIM
Associate Editor - Small Animal Internal Medicine, Nutrition
The Ohio State University
Steven Friedenberg, DVM, PhD, DACVECC
Associate Editor - Small Animal Internal Medicine (Genetics, Genomics)
University of Minnesota
Anthony Mutsaers, DVM, PhD, DACVIM
Associate Editor - Oncology
University of Guelph
John Farrelly, DVM, DACVIM, DACVR
Associate Editor - Oncology
Blue Pearl Veterinary Partners

**ACVIM Board of Regents Officers**

Joerg Steiner, DMV, PhD, DACVIM, DECVIM-CA
Board Chair
Sarah Gillings, DVM, MS, DACVIM
President
Bill Tyrrell, DVM, DACVIM
Treasurer

**Ex-Officio Member of the ACVIM Board of Regents**

Jane Sykes, BVSc (Hons), PhD, MBA, DACVIM
Immediate Past Board Chair

**The Editorial and ACVIM Offices are located at:**

116 Inveress Drive East, Suite 375, Englewood, CO 80112

**Telephone:** 303-231-9933 or 800-245-9081

**Editorial Email:** JVIM@ACVIM.org.
**ACVIM Email:** ACVIM@ACVIM.org.

The Journal is available online at www.wileyonlinelibrary.com/journal/jvim.

**ACVIM Board of Regents Members**

Brent Credille, DVM, PhD, DACVIM
Member At-Large
Tracy Hill, DVM, DACVIM, PhD, DECVIM-CA
Member At-Large
Roger A. Hostutler, DVM, MS, DACVIM
President, Small Animal Internal Medicine
Chris Sanchez, DVM, PhD, DACVIM
President, Large Animal Internal Medicine
Steven L. Rosenthal, DVM, DACVIM
President, Cardiology
Joan R. Coates, DVM, MS, DACVIM
President, Neurology
Chad Johannes, DVM, DACVIM
President, Oncology
Shannon Carter, EdD, CAE
Chief Executive Officer

// Journal of Veterinary Internal Medicine

Open Access

The Official Journal of the American College of Veterinary Internal Medicine,
the European College of Veterinary Internal Medicine - Companion Animals, the European College of Veterinary Neurology, and the European College of Equine Internal Medicine

Wiley Open Access articles posted to repositories or websites are without warranty from Wiley of any kind, either express or implied, including, but not limited to, warranties of merchantability, fitness for a particular purpose, or non-infringement. To the fullest extent permitted by law Wiley disclaims all liability for any loss or damage arising out of, or in connection with, the use of or inability to use the content.

**Open Access and Copyright**

All articles published by *Journal of Veterinary Internal Medicine* are fully open access: immediately freely available to read, download and share. All articles are published under the terms of a Creative Commons license. Copyright on any research article published in the *Journal of Veterinary Internal Medicine* is retained by the author(s).

Further information about open access licenses and copyright can be found at: https://authorservices.wiley.com/author-resources/Journal-Authors/licensing/licensing-info-faqs.html#4. Information on the licenses used by *Journal of Veterinary Internal Medicine* can be found at: www.wileyonlinelibrary.com/journal/jvim

**Disclaimer**

The Publisher, the American College of Veterinary Internal Medicine and Editors cannot be held responsible for any errors in or any consequences arising from the use of information contained in this journal. The views and opinions expressed do not necessarily reflect those of the Publisher, the American College of Veterinary Internal Medicine or Editors, neither does the publication of advertisements constitute any endorsement by the Publisher, the American College of Veterinary Internal Medicine, Editors, or Authors of the products advertised.

For submission instructions and all other information visit: www.wileyonlinelibrary.com/journal/jvim
